# Differential Evolution of *Achromobacter* spp. Isolates in Upper and Lower Airways of People with Cystic Fibrosis

**DOI:** 10.3390/pathogens15050452

**Published:** 2026-04-22

**Authors:** Laura Veschetti, Angela Sandri, Giulia Maria Saitta, Marzia Boaretti, Paola Melotti, Cristina Cigana, Alessandra Bragonzi, Maria M. Lleò, Giovanni Malerba, Caterina Signoretto

**Affiliations:** 1 Infections and Cystic Fibrosis Unit, Division of Immunology, Transplantation and Infectious Diseases, IRCCS San Raffaele Scientific Institute, 20132 Milano, Italy; veschetti.laura@hsr.it (L.V.); cigana.cristina@hsr.it (C.C.); bragonzi.alessandra@hsr.it (A.B.); 2 Vita-Salute San Raffaele University, 20132 Milano, Italy; 3 General and Upper GI Surgery, Azienda Ospedaliera Universitaria Integrata Verona, 37126 Verona, Italy; 4 Diagnostic and Public Health Department, University of Verona, 37134 Verona, Italy; giuliamaria.saitta@univr.it (G.M.S.); marzia.boaretti@univr.it (M.B.); caterina.signoretto@univr.it (C.S.); 5 Clinical Genetics Unit, Azienda Ospedaliera Universitaria Integrata Verona, 37126 Verona, Italy; paola.melotti@aovr.veneto.it; 6 Cystic Fibrosis Centre, Azienda Ospedaliera Universitaria Integrata Verona, 37126 Verona, Italy; 7 GMLab, Department of Surgical Sciences, Dentistry, Gynaecology and Paediatrics, University of Verona, 37134 Verona, Italy; giovanni.malerba@univr.it

**Keywords:** *Achromobacter*, cystic fibrosis, lung, bacterial adaptation, hypermutation, virulence, antibiotic resistance, airway reservoir

## Abstract

*Achromobacter* spp. are opportunistic pathogens in people with cystic fibrosis (PwCF), yet the role of the upper airways in their persistence and adaptation remains poorly understood. We investigated whether the sinonasal compartment may act as reservoir and evolutionary niche for *Achromobacter* spp. during airway infection. Twenty-two isolates obtained from paired nasal lavage and sputum samples of seven PwCF were analysed by whole-genome sequencing. Within each PwCF, identical clone types were detected in both airway compartments, supporting bacterial exchange between upper and lower airways. Despite clonal relatedness, substantial genomic diversification was observed between paired isolates. Genomic signatures indicative of elevated mutation rates were detected in a high number of isolates (73%) and in both airway compartments, highlighting widespread genomic diversification across the respiratory tract. Mobilome analysis revealed compartment-specific variations in insertion sequences, prophages, and integrative elements, suggesting genome plasticity. Additionally, mutation in an aspartate kinase gene was consistently associated with loss of biofilm formation in vitro, highlighting a potential link between this pathway and biofilm phenotype. Overall, our findings indicate that upper and lower airways represent interconnected but partially independent ecological niches where *Achromobacter* populations can diverge during colonization, supporting the view that both compartments contribute to their persistence and evolution in CF airways.

## 1. Introduction

According to the united airways concept, the respiratory tract functions as a continuous ecological system in which the upper and lower airways are closely interconnected. This concept has been particularly relevant in cystic fibrosis (CF), where several studies have demonstrated a strong correlation between the microbiome of the sinonasal compartment and that of the lungs. In children with CF, *Staphylococcus aureus* and *Haemophilus influenzae* are frequently detected in the upper airways, whereas *Pseudomonas aeruginosa* tends to become increasingly prevalent with age, reflecting the microbial patterns often observed in the lower respiratory tract [1,2,3]. Moreover, several studies found concordance between upper and lower airways microbiota in people with CF (PwCF), and bacteria isolated from the two sites in the same individual often have the same clone type [4,5,6,7]. These findings support the hypothesis that the upper airways function as a reservoir where bacteria can adapt and be aspirated to the lungs and vice versa. Further support for this concept has emerged from more recent microbiome-based studies, which have demonstrated the long-term persistence of CF pathogens in the sinonasal compartment and the low diversity of microbial communities inhabiting these niches [8,9,10]. In many PwCF, the sinuses harbor dominant pathogens over extended periods of time, suggesting that the upper airways constitute a relatively stable ecological niche that may facilitate bacterial persistence and adaptation [8,9].

Chronic or recurrent colonization of the upper airways might act as a potential source of bacteria that can periodically re-colonize the lower respiratory tract. Evidence supporting this hypothesis has been provided by studies investigating the evolutionary dynamics of *P. aeruginosa* in the sinonasal compartment. Hansen and colleagues [4] showed that the colonization events with *P. aeruginosa* in the sinuses may provide extended opportunities for evolution of the bacteria towards phenotypes with a higher potential of causing chronic lung infections as well. Sinus isolates displayed phenotypic and genotypic changes similar to those commonly observed during chronic lung infections in CF, indicating that important adaptive processes may occur in the upper airways before the pathogen establishes persistent colonization of the lungs.

In recent years, the landscape of CF airway infection has been profoundly reshaped by the introduction of highly effective Cystic Fibrosis Transmembrane Conductance Regulator (CFTR) modulator therapies. These treatments improve mucociliary clearance and reduce airway inflammation, frequently resulting in decreased sputum production and lower bacterial densities in respiratory samples [11]. As a consequence, the microbiological surveillance of respiratory pathogens has become more challenging, as many patients receiving CFTR modulators no longer expectorate sputum routinely [12]. Although the culture-based detection of classical CF pathogens has declined in the modulator era, several studies have shown that bacteria such as *P. aeruginosa* and other opportunistic pathogens, including *Achromobacter* spp., often persist at low abundance and may remain detectable by molecular methods despite negative cultures [13,14]. Notably, recent evidence indicates that while the overall bacterial load of *Achromobacter* spp. may decrease under highly effective CFTR modulator therapy; its prevalence does not appear to change substantially, suggesting continued persistence in a subset of patients despite treatment. This suggests that apparent microbiological clearance does not necessarily correspond to true eradication of bacterial populations from the respiratory tract. The reduced availability of sputum samples has also increased the use of alternative respiratory specimens, including throat or nasal swabs, for microbiological monitoring. However, concordance between pathogens detected in the upper and lower airways is often limited, indicating that these anatomical compartments may represent partially independent ecological niches [15]. Understanding the role of the upper airways as potential reservoirs of persistent bacteria is therefore increasingly important in the context of the evolving clinical landscape of CF.

Among emerging CF pathogens, species of the genus *Achromobacter* have gained increasing attention due to their ability to establish both intermittent and chronic infections in the lungs of PwCF, and to their persistence despite CFTR modulator therapies.

The clinical significance of *Achromobacter* spp. in CF is increasingly recognized, as chronic infection—particularly with *A. xylosoxidans*—has been associated with heightened airway inflammation, a higher frequency of pulmonary exacerbations, and increased hospitalization rates [13,14,16]. Its persistence is driven by a complex within-host evolutionary process characterized by the accumulation of pathoadaptive mutations and the frequent emergence of hypermutable lineages—caused by defects in DNA repair systems—which promote rapid clonal diversification [17,18,19,20,21,22,23]. While multidrug resistance (both innate and acquired) is a hallmark of the genus, its adaptation also involves metabolic shifts toward anaerobic survival and the modification of virulence factors, such as biofilm formation and immune-evasive LPS alterations [18,21,24]. Furthermore, the high genomic plasticity of *Achromobacter*, facilitated by an extensive mobilome, allows for the continuous acquisition of adaptive traits through horizontal gene transfer [25]. Despite growing knowledge about the evolutionary dynamics of *Achromobacter* spp. in the lower airways, little is known about their presence and adaptation in the upper respiratory tract. Given the similarities between *Achromobacter* spp. and other CF opportunistic pathogens such as *P. aeruginosa*, it is plausible that the sinonasal compartment may also serve as a reservoir for these bacteria, providing opportunities for adaptation and potentially facilitating repeated colonization of the lungs.

In this study, we investigated whether the upper airways may represent an important niche for the adaptation of *Achromobacter* spp. in PwCF and whether bacterial populations in the upper and lower airways undergo convergent or divergent evolutionary trajectories. Understanding the evolutionary dynamics of *Achromobacter* spp. across airway compartments may provide important insights into the mechanisms that enable bacterial persistence and may ultimately inform therapeutic strategies aimed at achieving more effective eradication of these opportunistic pathogens.

## 2. Materials and Methods

### 2.1. Samples Collection and Bacterial Identification

**Figure 1:**
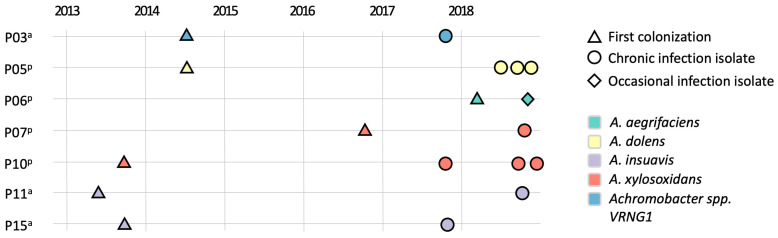
*Achromobacter* spp. isolates collection overview. The timeline shows the dates of collection of *Achromobacter* spp. isolates for each PwCF enrolled in the study, along with the date of the first colonization event (reported as clinical metadata and not included among the analyzed isolates). A minimum of one isolate and a maximum of three isolates were collected from 7 patients between 2017 and 2018. On the left, PwCF identification numbers are reported, followed by adult (a) or pediatric (p) status. On the right, shapes indicate infection type, and colors indicate *Achromobacter* species.

Twenty-two isolates were collected from paired sputum and nasal lavage samples of 7 patients followed at the CF Center of Verona between 2017 and 2018 and were identified as *Achromobacter* spp. by MALDI-TOF-MS (bioMerieux, Marcy-l’Étoile, France). At the time of sample collection, all patients were undergoing standard-of-care pharmacological treatment for CF, which primarily included inhaled antibiotics, bronchodilators and mucolytics, e.g., tobramycin/colistin, Beta-agonists and DNase, respectively; oral antibiotics, mainly ciprofloxacin, amoxicillin, sulfamethoxazole/trimethoprim, and IV cycles including tobramycin/amikacin and ceftazidime. None of the subjects was receiving CFTR modulator therapy. Additional information on PwCF and *Achromobacter* spp. isolates is reported in Table S1. Sampling timeline is shown in Figure 1. The study was conducted in accordance with the Declaration of Helsinki, and approved by the local Ethics Committee of the Centro Ricerca Clinica of Verona (protocol codes CRCFC026 approval date 26 July 2017 and CRCFC-CEPPO031 approval date 27 April 2016). According to the European Consensus criteria, infection was defined as chronic when at least three positive cultures over ≥6 months were obtained with at least a 1-month interval between samples [26]. Samples were collected approximately every 3 months for microbiological analysis. The classification of occasional and chronic infection was assessed using the information regarding all the *Achromobacter* spp. strains identified between 2013 and 2018. A minimum of 1 isolate and a maximum of 3 isolates per site from each infected patient were stored and then used in this study, providing a representative snapshot of the strains recovered at each time point. Patient identifiers were retained from our previous studies on the same cohort to ensure consistency and enable cross-referencing across datasets. These identifiers are used solely for anonymized tracking purposes and do not carry any clinical meaning.

### 2.2. Biofilm Formation Assay

Biofilm formation was assessed as previously described [24]. Bacterial strains were plated onto LB agar (Merck KGaA, Darmstadt, Germania) and grown at 37 °C for 24–48 h. A single colony was inoculated in Brain Heart Infusion medium (Merck KGaA, Darmstadt, Germania) and grown for 16 h at 37 °C with shaking. OD_600_ was measured, cultures were diluted to 0.1 OD/mL and 200 µL/well were incubated in a 96-well plate for 24 h at 37 °C. Wells were washed with saline solution and stained with 0.1% crystal violet solution for 15 min, then rinsed, washed with water and air dried. After 30 min of incubation with 30% acetic acid at 37 °C, absorbance at 550 nm was measured. Isolates were defined as forming or not forming biofilm as proposed by Stepanovic and colleagues [27].

### 2.3. Whole Genome Sequencing

All collected isolates were subjected to whole genome sequencing at the Technological Platform Centre of the University of Verona, as previously described [25,28]. The resulting sequencing data were deposited in the NCBI SRA database under the project accession numbers PRJEB40979 (sputum isolates) and PRJEB109938 (nasal lavage isolates). Genomic DNA was extracted using the QIAamp DNA Blood Mini Kit (Qiagen, Milan, Italy). DNA quantity and quality were assessed using a NanoDrop 2000 spectrophotometer (Thermo Fisher Scientific, Wilmington, DE, USA) and a Fragment Analyzer System (Agilent Technologies, Santa Clara, CA, USA). Sequencing libraries were prepared with the KAPA PCR-free kit (Roche Sequencing Solutions, Pleasanton, CA, USA) and sequenced on an Illumina NextSeq500 platform (Illumina, Hayward, CA, USA), generating 150 bp paired-end reads with a mean yield of 5,595,337 reads per sample and an average coverage depth of 186×.

### 2.4. Genomic Data Processing, Phylogenomic Analysis, and In Silico Strain Typing

Read quality control, *de novo* genome assembly, and genome annotation were carried out following the same procedures described in our previous study [22]. Briefly, raw reads were quality-checked using FastQC v0.12.1 [29], and adapter and quality trimming were performed with Trimmomatic v0.39 [30]. *De novo* genome assemblies were generated using SPAdes v3.15.5 [31]. Assembly quality was assessed through read mapping and coverage statistics, and genomes were subsequently annotated using Prokka v1.14.6 [32]. Average nucleotide identity (ANI) values between all sequenced isolates and all available *Achromobacter* spp. genomes (n = 142; NCBI RefSeq database) were calculated using fastANI v1.33 [33] to assign species-level classification. Isolates were considered to belong to the same species when ANI values were ≥95%. Based on ANI results, the following reference genomes for each identified species were included in subsequent analyses: *A. aegrifaciens* GCF_000165835.1; *A. dolens* GCF_001051055.1; *A. insuavis* GCF_001558755.2; *A. xylosoxidans* GCF_001457475.1. Phylogenomic analyses were conducted using the parsnp v1.2 tool of the Harvest-OSX64-v1.1.2 suite [34]. In silico Multi-Locus Sequence Typing (MLST) analysis was conducted using mlst v2.23.0 [35] following the *Achromobacter* MLST scheme [36].

### 2.5. Genomic Characterization of the Isolates: Virulence Factors, Antibiotic Resistance and Mobile Genetic Elements

To determine the presence of virulence factor, antibiotic resistance genes, and plasmids, the *de novo* assemblies were screened using Abricate v1.0.1 [35] to search against the VFDB (n = 2597 nucleotide sequences) [37], MEGARes (n = 6635 nucleotide sequences) [38], Refinder (n = 3077 nucleotide sequences) [39], CARD (n = 2631 nucleotide sequences) [40], and Plasmidfinder (n = 460 nucleotide sequences) [41] databases. Hits were retained only if they met the thresholds of ≥50% gene coverage and ≥90% nucleotide identity. Prophage sequences were identified and annotated using the Phage Search Tool Enhanced Release (PHASTER) [42], based on sequence similarity. Only prophages classified as intact by the tool were included in subsequent analyses. The presence of integrative and conjugative elements (ICEs), integrative and mobilizable elements (IMEs), and cis-mobilizable elements was assessed using the online version of ICEfinder, based on the ICEberg database [43]. Insertion sequences (ISs) were identified using ISfinder [44]. Given that analyses were performed on draft genome assemblies derived from short-read sequencing, the identification of mobile genetic elements was limited to presence/absence signals based on sequence similarity, and their complete structural reconstruction could not be resolved. Annotations of the identified mobile genetic elements were manually inspected to assess the presence of antimicrobial resistance genes, virulence factors, mismatch repair genes, proteins associated with mobile element stability and function, and toxin-antitoxin systems. Heatmaps for data visualization were generated using the pheatmap package v1.0.13 in R v4.4.1.

### 2.6. Variant Calling and Mutator Genes Analysis

To assess longitudinal genomic changes among isolates, quality-filtered reads were mapped to the appropriate reference genome using Bowtie2 v2.3.4.1 [45] and variant calling was performed using the Snippy v4.6.0 pipeline (available at: https://github.com/tseemann/snippy accessed on 5 July 2023). Analyses included all genome sequences obtained from the same PwCF and belonging to the same clone type, using the closest available reference genome for comparison. The use of a species-level reference genome, in the absence of a personalized reference genome for each PwCF, may reduce the sensitivity for detecting large structural variants. The genetic basis of hypermutation was investigated using whole-genome sequencing data by analyzing genes previously reported to be associated with this phenotype. The following mutator genes were examined based on evidence from the literature: *mutL*, *mutS*, *pfpI*, super oxide dismutase, *radA*, *radC*, *rad50*, *uvrA*, *uvrB*, *uvrC* and *uvrD* [23]. To evaluate the presence of hypermutator strains, multiple parameters were assessed, including the occurrence of high-impact and deleterious missense mutations in mutator genes, the transition/transversion ratio, and both the overall and per-site mean yearly variant rates.

### 2.7. Pangenome and Gene-Trait Association Analyses

Pangenome analysis was conducted to identify genes potentially associated with biofilm production. Annotated genome files in GFF3 format were used as input for Roary v3.11.2 [46] to construct the pangenome of all sequenced isolates. Roary was run with parameters enabling core gene alignment using PRANK and fast alignment with MAFFT, generating presence/absence matrices for all genes across isolates. To explore potential gene-trait associations with the biofilm-producing phenotype, Scoary v1.6.16 [47] was applied using the pangenome gene presence/absence matrix and a trait file encoding the phenotype of each isolate. Given the inclusion of multiple isolates per PwCF and the resulting non-independence of samples, this analysis was performed in an exploratory and hypothesis-generating framework rather than as a conventional genome-wide association study. Gene-trait associations were evaluated across the phylogenetic tree of accessory genes, and statistical significance was determined using 10,000 permutations with population structure correction.

## 3. Results

### 3.1. Collection Overview and Phylogenomic Characterization of Achromobacter spp. Isolates

A total of seven PwCF (three adults and four pediatric) colonized with *Achromobacter* spp. in both upper and lower airways were included in the study. From these individuals, we detected between one and three paired isolates from sputum and nasal lavage over a one-year period, resulting in a total of 22 isolates (11 from each site). The timeline of isolate collection and first colonization events for each PwCF is shown in Figure 1, while detailed individuals and isolate information is reported in Table S1. This sampling strategy, limited to seven patients and a maximum of three isolates per site, is intended to provide a comparative snapshot of *Achromobacter* spp. evolution; we acknowledge that the restricted cohort size and sampling depth may not fully capture the complete range of within-host diversity or the broader dynamics across diverse CF populations.

**Figure 2:**
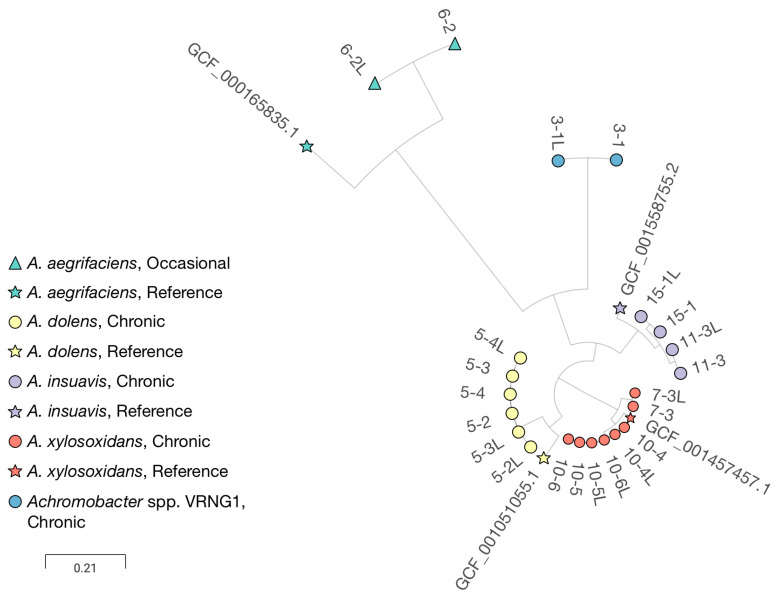
Phylogenomic tree inferred from core genome SNPs of *Achromobacter* spp. isolates included in the study. Colors indicate the *Achromobacter* species assigned by average nucleotide identity (ANI) analysis, while symbol shapes denote the isolate type. The scale bar represents genomic distance based on the number of core-genome SNPs. In the isolate IDs the first number corresponds to each PwCF, the second number indicates the collection time point, “L” identifies strains isolated from nasal lavage.

All isolates were subjected to whole genome sequencing, and sequencing metrics, assembly statistics, and annotation details are provided in Tables S2 and S3. Multiple *Achromobacter* species were identified among the cohort, including *A. xylosoxidans* (n = 2), *A. insuavis* (n = 2), *A. dolens* (n = 1), *A. aegrifaciens* (n = 1), and one isolate that could not be assigned to any known species based on ANI analysis. Following our previous nomenclature [28], this isolate is referred to as a Verona non-affiliated genogroup 1 (VRNG1), highlighting the genetic diversity and ongoing taxonomic refinement within the genus. According to European Consensus criteria, all infections were classified as chronic except for a single occasional infection (P06, *A. aegrifaciens*). In all PwCF with longitudinal isolates available, the same species and strain type were consistently detected in both the upper and lower airways. Phylogenomic analysis based on core-genome SNPs assisted species-level assignments and revealed clustering of isolates by PwCF rather than by sampling site (Figure 2), supporting the notion that each patient harbors a distinct, persistent strain in both the upper and lower airways.

### 3.2. Genomic Profiling of Virulence, Antibiotic Resistance, and Mobile Genetic Elements

**Figure 3:**
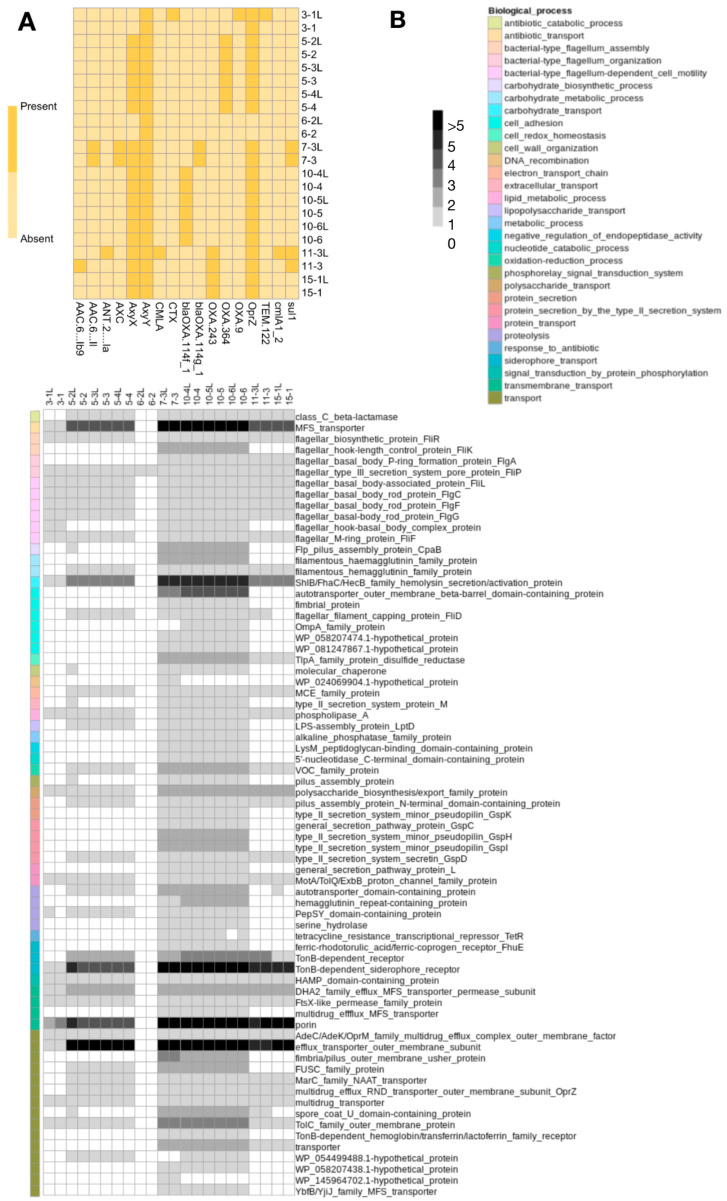
Genomic characterization of *Achromobacter* spp. isolates: virulence factors and antibiotic resistance genes. (**A**) Heatmap showing antibiotic resistance genes identified by screening genome assemblies against the CARD, MEGARes, and ResFinder databases. Gene presence is indicated using a yellow color scale (darker color indicates gene presence). (**B**) Heatmap showing the presence of virulence factor genes identified using the VFDB database. Gene presence is indicated in grayscale, with darker shades corresponding to a higher number of gene copies. The corresponding Gene Ontology (GO) biological process annotations are shown in the colored annotation column. Isolates labeled “L” correspond to strains obtained from nasal lavage samples.

The genomic profiles of *Achromobacter* spp. isolates were characterized to investigate the distribution of virulence factors, antibiotic resistance genes, and mobile genetic elements across isolates from upper and lower airways. Overall, the repertoire of antibiotic resistance genes was largely conserved between paired isolates from the two anatomical sites. Differences in resistance gene content were observed only in two patients (P03 and P11) (Figure 3A). In contrast, variations in virulence-associated genes were more frequently detected, involving isolates from four patients (P03, P05, P10, and P15) (Figure 3B). Among longitudinal isolates, additional variation in virulence gene profiles was observed in upper airway strains. In particular, one nasal lavage isolate (5-2L) displayed a broader set of virulence-associated genes compared with the paired lung isolate collected at the same time point. However, subsequent isolates suggested either loss of these genes or replacement of this subpopulation by the lower airway strain.

**Figure 4:**
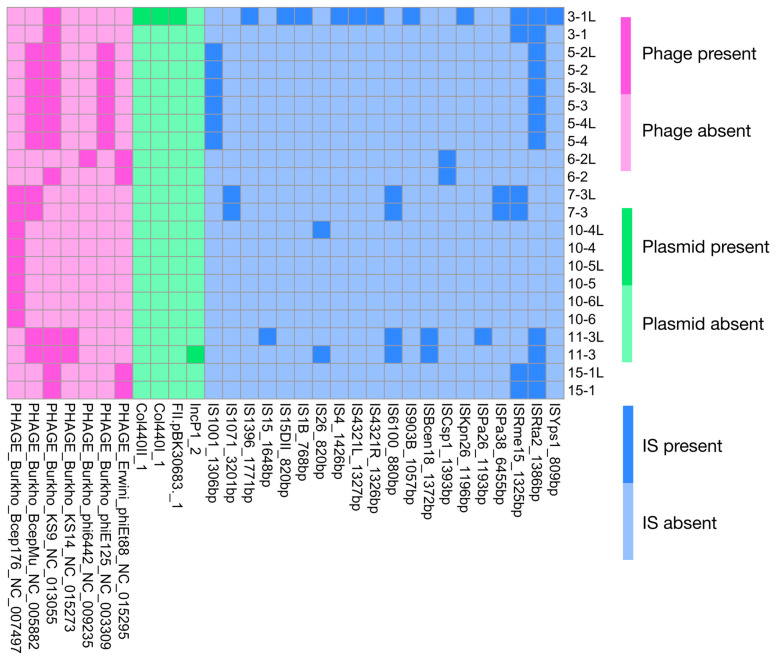
Distribution of mobile genetic elements across *Achromobacter* spp. isolates. Heatmap showing the presence of prophages, plasmids, and insertion sequences (IS). Prophages identified using PHASTER are shown in purple, plasmids detected using Abricate against the PlasmidFinder database are shown in green, and insertion sequences identified using ISfinder are shown in blue. Darker colors indicate the presence of the corresponding element. Only prophages classified as intact were included in the analysis. Isolates labeled “L” correspond to strains obtained from nasal lavage samples.

We further explored the distribution of mobile genetic elements, including insertion sequences (ISs), prophages, integrative and conjugative elements (ICEs), and integrative and mobilizable elements (IMEs) (Figure 4, Tables S4–S6). Most of the identified prophages (six out of seven) showed sequence similarity with phages originally described in *Burkholderia* spp., another common CF-associated pathogen. Differences in at least one class of mobile genetic element between paired upper and lower airway isolates were detected in five out of seven patients. In three of these patients (P03, P10, and P11), variations involved both insertion sequences and ICEs. Notably, one nasal lavage isolate (3-1L) contained a particularly high number of insertion sequences (n = 11), whereas the paired lower airway isolate carried only a few. Longitudinal sampling also revealed mobilome variability over time. In patient P10, changes in IS and ICE content were observed in upper airway isolates, while in patient P05 ICE variations occurred across isolates from both anatomical sites.

### 3.3. Hypermutation and Genomic Adaptation Across Airway Compartments

**Figure 5:**
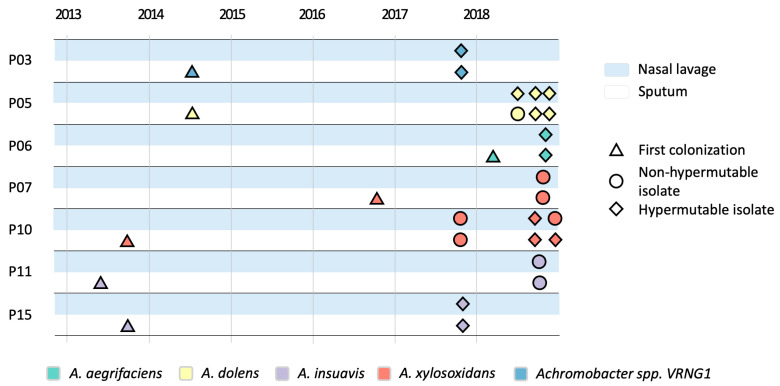
Overview of *Achromobacter* spp. isolates in relation to sample origin and hypermutation status. The timeline shows the dates of collection of *Achromobacter* spp. isolates for each PwCF enrolled in the study, along with the date of the first colonization event (reported as clinical metadata and not included among the analyzed isolates). Background color indicates sample type (light blue: nasal lavage; white: sputum). Symbol shapes indicate isolate hypermutability status and colors represent *Achromobacter* species.

Genome-wide variant analysis confirmed that isolates recovered from the upper and lower airways of each PwCF belonged to the same clone type (max SNP distance = 291; genomes from different clone types differ by >10.000), indicating the presence of a dominant lineage shared across airway compartments. Longitudinal variant analysis against the corresponding reference genomes revealed elevated mutation rates in most isolates (Table S7). In particular, many strains displayed both increased mean yearly variant accumulation rates and high transition/transversion (Ts/Tv) ratios, features commonly associated with hypermutator phenotypes resulting from defects in DNA repair systems. Hypermutation is an adaptive mechanism well-studied in *P. aeruginosa* and we demonstrated that it can occur also during *A. xylosoxidans* colonization of CF airways [22]. To investigate the genetic basis of hypermutation, we analyzed a set of genes previously associated with mutator phenotypes, including *mutL*, *mutS*, *pfpI*, superoxide dismutase, *radA*, *radC*, *rad50*, *uvrA*, *uvrB*, *uvrC*, and *uvrD* [17]. The copy number of these genes across isolates is reported in Table S8, while mutations affecting mutator genes are summarized in Table S9. Based on the combined evaluation of mutation rates, Ts/Tv ratios, and the presence of high-impact or deleterious missense mutations in mutator genes, 16 out of 22 isolates (73%) displayed genomic features consistent with hypermutator phenotypes. These included eight isolates from nasal lavage samples and eight from sputum. Hypermutator strains were detected in all patients except one (P07). Different patterns of hypermutation emergence were observed across patients (Figure 5). In four patients (P03, P06, P11, and P15), hypermutator strains were detected simultaneously in isolates from both the upper and lower airways. In contrast, two patients showed site-specific temporal dynamics. In patient P05, genomic features consistent with a hypermutator phenotype were first observed in upper airway isolates and later in the lower airway strains. Conversely, in patient P10 hypermutation emerged in isolates from both sites but was subsequently lost in the upper airway population, possibly reflecting a fitness cost associated with the hypermutator state in that niche.

### 3.4. Exploratory Gene-Trait Analysis Identifies a Candidate Determinant Potentially Associated with Biofilm Formation

**Figure 6:**
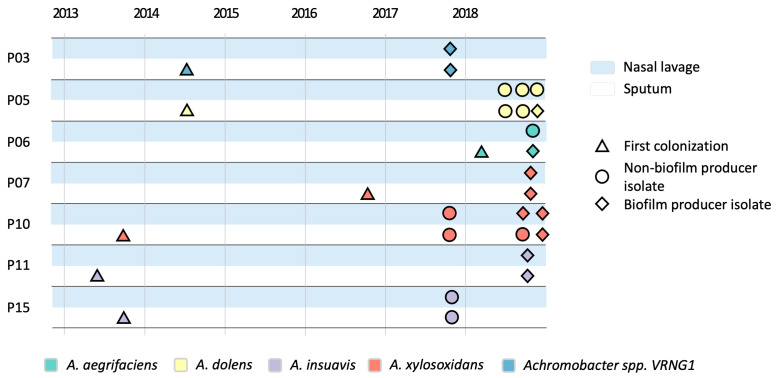
Overview of *Achromobacter* spp. isolates in relation to sample origin and biofilm production. The timeline shows the dates of collection of *Achromobacter* spp. isolates for each PwCF enrolled in the study, along with the date of the first colonization event (reported as clinical metadata and not included among the analyzed isolates). Background color indicate sample type (light blue: nasal lavage; white: sputum). Symbol shapes indicate biofilm production capabilities and colors represent *Achromobacter* species.

In most paired isolates from the same patient, the biofilm phenotype was conserved between upper and lower airway strains, suggesting that biofilm formation is mainly clone-specific rather than strictly niche-specific (Figure 6, Table S10). For example, isolates from patients P03, P07, P11, and P15 exhibited the same ability (or inability) to form biofilm regardless of the anatomical site. However, a few paired isolates showed discordant phenotypes, indicating that local diversification within a single host may occur. In individuals P5 and P6, the lung isolates produced biofilm while the paired nasal lavage isolate did not. Conversely, for P10, a nasal isolate formed biofilm while the corresponding lung isolate was negative and in the following sampling both isolates produced biofilm. To explore potential genetic factors associated with biofilm formation, we performed pangenome and gene-trait association analyses (Table S11). Given the limited number of isolates and the non-independence of samples derived from the same patients, this analysis was conducted in an exploratory, hypothesis-generating framework and was not intended to identify robust gene–phenotype associations. Consistent with these limitations, no statistically significant associations were identified. Nevertheless, we examined the highest-ranking candidate genes as a prioritization step for further investigation. Among these, the gene encoding aspartate kinase (AK) emerged as a plausible determinant of the biofilm phenotype. Sequence inspection revealed two variants: a full-length gene (1265 bp) and a truncated version (1229 bp) lacking part of the predicted ATP-binding domain. Comparison with phenotypic biofilm data showed a strong association between gene variant and biofilm formation. Among isolates carrying the truncated gene, 91% (10/11) were unable to form biofilm in vitro, whereas all isolates harboring the full-length gene produced biofilm except for 6-2L isolate (Table S10). These results suggest that variation in the AK gene may contribute to phenotypic differences in biofilm formation among *Achromobacter* spp. isolates and, in some cases, may be associated with patterns of diversification observed between airway compartments.

## 4. Discussion

The present study investigated whether the upper airways may constitute a relevant niche for the adaptation of *Achromobacter* spp. in PwCF and whether this compartment could potentially contribute to the persistence of lung infection. By analysing paired isolates from nasal lavage and sputum samples collected from the same patients, we observed that identical clonotypes were consistently present in both sites, while genomic and phenotypic differences emerged between isolates from the two compartments. These findings support the hypothesis that the upper and lower airways represent interconnected but partially independent ecological niches in which bacterial populations can diverge during colonization.

The subjects were colonized with different *Achromobacter* species. Notably, the only occasional infection in our cohort was caused by *A. agrifaciens*, consistent with its reportedly lower virulence and adaptation potential in the CF airway environment [28]. The observation that the same *Achromobacter* species and clone types were detected simultaneously in the upper and lower airways of each patient is consistent with the concept of the “united airways”, according to which microbial populations can migrate between different compartments of the respiratory tract. Similar concordance between upper and lower airway isolates has been extensively reported for *P. aeruginosa* in PwCF, where the paranasal sinuses have been proposed as a reservoir for lung infection [4,5]. Previous work demonstrated that *P. aeruginosa* populations colonizing the sinuses can undergo extensive adaptation before establishing chronic infection in the lungs [4]. More recent microbiome studies have further highlighted the persistence of CF pathogens within the sinonasal niche and their potential role in sustaining airway infection despite antibiotic therapy [8,9,10]. Our results extend this concept to *Achromobacter* spp., suggesting that similar evolutionary dynamics may occur for this emerging CF pathogen.

A major finding of this study is the high prevalence of hypermutator phenotypes among the analyzed isolates. Hypermutation was detected in the majority of strains (73%), and notably occurred in isolates from both the upper and lower airways. It should be noted that our analysis focused on a predefined set of mutator genes based on current literature and may not capture additional or recently identified determinants, nor regulatory mechanisms such as promoter mutations or epigenetic factors that could also contribute to this phenotype. Hypermutability is a well-known adaptive mechanism in chronic CF infections and has been widely described in *P. aeruginosa*, where defects in DNA mismatch repair genes promote rapid diversification and accelerate the accumulation of adaptive mutations conferring a selective advantage under antibiotic treatment [17,48]. Similar mechanisms have been reported for *Achromobacter* spp., where genomic studies have shown that long-term airway colonization is accompanied by pathoadaptive mutations and genetic diversification [19,20,22]. The presence of hypermutator strains in both airway compartments suggests that strong selective pressures operate throughout the respiratory tract of PwCF, likely including antibiotic exposure, host immune responses, and nutrient limitations. Interestingly, in some patients hypermutation appeared to arise first in the upper airways and subsequently in the lower airways, whereas in others it was lost in one compartment over time. These observations suggest that adaptive trajectories may differ between niches and that hypermutation may confer advantages only under specific environmental conditions. Similar compartment-specific evolutionary patterns have been reported for *P. aeruginosa*, where independent adaptation processes can occur in different regions of the airway system [4]. However, important biological differences between *P. aeruginosa* and *Achromobacter* spp. should be considered, and such parallels remain to be fully validated in *Achromobacter*. Crucially, the sinus environment differs from the lung in several critical physiological and pharmacological aspects that shape bacterial evolutionary strategies. These include lower oxygen availability—often resulting in microaerophilic or anaerobic niches, distinct mucociliary clearance rates, and variations in immune pressure [49,50,51]. Furthermore, antibiotic exposure levels vary significantly between these compartments; while the lower airways are the primary target of aggressive eradication protocols and high-concentration inhaled therapies, the sinuses frequently function as protected niches where drug penetration is limited by the anatomical structure of the paranasal cavities [51,52]. This disparity in selective pressure may allow the upper airways to serve as a reservoir where bacterial populations can persist and adapt independently, potentially reseeding the lungs following the apparent success of lower-airway-directed treatments.

Beyond point mutations, we also observed substantial variation in the mobilome among isolates from the same patients. Differences in insertion sequences, prophages and integrative elements were detected between upper and lower airway isolates in most patients. Notably, most prophages identified in our isolates were originally described in *Burkholderia* species, another opportunistic pathogen frequently found in CF airways. Horizontal gene transfer mediated by bacteriophages and other mobile genetic elements is increasingly recognized as an important driver of bacterial evolution in CF infections, contributing to the acquisition of virulence factors, antibiotic resistance determinants and metabolic capabilities [53]. Previous genomic studies have shown that *Achromobacter* populations in CF lungs possess highly dynamic accessory genomes enriched in mobile elements [25]. Our findings suggest that these genetic exchanges may also occur within the sinonasal compartment, potentially facilitating rapid adaptation to local environmental conditions.

Although variations in antibiotic resistance genes were relatively limited, differences in virulence gene content from the two compartments were observed in several patients, supporting the existence of niche-specific adaptation. Variations in virulence gene content were detected in several patients and were particularly evident among longitudinal isolates from the upper airways. These findings indicate that the sinus environment may promote diversification of virulence-associated traits, which could subsequently influence bacterial fitness during lung colonization. A similar phenomenon has been described for other CF pathogens, where sinus populations serve as reservoirs of genetically diverse subpopulations that can reseed the lungs over time [4].

An additional observation concerns the association between biofilm formation and the integrity of the aspartate kinase gene identified in several isolates. Although this pattern may reflect lineage-specific variation (given the limited sample size and the strong clonal structure of the dataset) rather than causal determinants of biofilm formation, in our dataset most isolates lacking a complete gene sequence were unable to form biofilm in vitro, whereas strains carrying the intact gene retained this phenotype. Biofilm formation is traditionally considered an important virulence factor in chronic airway infections because it can protect bacteria from host immune responses and antibiotic treatments. In *P. aeruginosa*, metabolic pathways including amino acid biosynthesis have been implicated in biofilm regulation and persistence within the CF airway environment [54]. However, increasing evidence suggests that biofilm production may not be essential for *Achromobacter* spp. persistence in CF airways. For instance, studies analysing collections of CF isolates have shown that only a subset of strains form strong biofilms and that biofilm production does not necessarily correlate with infection type or persistence in the host [24,55]. These findings indicate that *Achromobacter* spp. may rely on alternative strategies for long-term survival in the CF lung, including antibiotic resistance, metabolic adaptation and other virulence traits [24]. In this context, although the functional role of aspartate kinase in *Achromobacter* spp. remains to be clarified, our observations suggest that alterations in this pathway could influence biofilm production without necessarily determining the ability of these bacteria to establish persistent airway colonization.

Taken together, our results indicate that *Achromobacter* populations in PwCF can undergo adaptive diversification across different airway compartments. While clonal relatedness confirms frequent exchange between upper and lower airways, the presence of compartment-specific mutations, mobilome variations and phenotypic differences suggests that these niches can drive independent evolutionary trajectories. These findings support the hypothesis that the upper airways may contribute to bacterial persistence and provide a site where adaptive diversification can occur alongside the lower airways.

From a clinical perspective, these observations may have important implications for the management of *Achromobacter* spp. infections in CF. In clinical practice, bacterial eradication following aggressive antibiotic therapy is generally defined by the absence of the pathogen in subsequent sputum cultures. This interpretation implicitly assumes that the pathogen has been eliminated from the entire respiratory tract and that any later detection represents acquisition of a new environmental strain. However, several studies have demonstrated that pathogens persisting in the upper airways can reseed the lungs after antibiotic treatment [8,9,10]. For instance, approximately in 25% of cases *P. aeruginosa* causes re-colonization with the same clone type involved in previous infections [56,57,58]. Such observations confirm that bacteria persist in protected niches within the airways despite apparent eradication from the lungs. Nonetheless, current eradication strategies primarily target lower airway infections, often neglecting the potential role of sinonasal reservoirs. Failure to eliminate bacterial populations from the sinuses may therefore contribute to recurrent colonization events and complicate eradication efforts. Addressing infections in both compartments may be important for achieving more effective long-term control of airway pathogens in PwCF.

This study has some limitations that should be considered. The number of patients analysed was relatively small and isolates were collected over a limited time period. Moreover, the analysis of a limited number of isolates per sample may not fully capture the within-host population diversity, potentially overlooking coexisting subpopulations. Although our results reveal important patterns of bacterial evolution, larger longitudinal studies are required to confirm these findings and determine whether similar dynamics occur across broader CF populations. In addition, functional validation of the identified genetic variants will be necessary to clarify their role in virulence, antibiotic resistance, and persistence. Furthermore, biofilm assay performed under static conditions and relying on a binary phenotype classification may miss clinically relevant variability and adaptation, thus warranting cautious interpretation of in vitro-identified determinants in the context of CF airway-specific selective pressures. Future research should aim to better characterize the ecological interactions between upper and lower airway microbiota and to determine how these communities influence the evolution of opportunistic pathogens. Integrating longitudinal genomics with transcriptomic and phenotypic analyses may provide deeper insight into the mechanisms underlying bacterial adaptation within the CF airway environment. Moreover, investigating how new CFTR modulator therapies influence microbial populations in both airway compartments may help clarify whether these treatments alter evolutionary trajectories.

## 5. Conclusions

Our findings support the concept that the upper airways represent an important niche for *Achromobacter* spp. adaptation in PwCF. The coexistence of clonally related yet genetically divergent populations between upper and lower airway compartments suggests ongoing evolutionary processes that may influence the establishment and persistence of chronic lung infection. A better understanding of these dynamics could help inform more comprehensive therapeutic strategies targeting both compartments of the respiratory tract.

## Data Availability

The genomic sequences analyzed in this study are openly available in NCBI SRA database within project numbers PRJEB40979 (sputum isolates) and PRJEB109938 (nasal lavage isolates).

## Supplementary Materials

The following supporting information can be downloaded at: https://www.mdpi.com/article/10.3390/pathogens15050452/s1. Table S1: PwCF and *Achromobacter* spp. isolates information; Table S2: Summary of read quality filtering and mapping statistics for each isolate; Table S3: Summary statistics of the whole-genome sequencing assemblies of the isolates included in this study; Table S4: Summary of mobile genetic elements identified in the genomes of the isolates analyzed in this study; Table S5: Distribution of genomic features associated with antimicrobial resistance, virulence, genome stability, and horizontal gene transfer across ICEs and IMEs; Table S6: Distribution of genomic features associated with antimicrobial resistance, virulence, genome stability, and horizontal gene transfer across phages; Table S7: Summary of genomic variants identified in the longitudinal and baseline isolates from PwCF; Table S8: Number of mutator genes identified in the genomes of the isolates analyzed in this study; Table S9: Summary of high-impact and missense mutations in mutator genes across the isolates analyzed in this study; Table S10: Quantification of biofilm formation by the analyzed isolates; Table S11: Gene-trait association analysis for biofilm formation in *Achromobacter* spp. isolates.

## Abbreviations

The following abbreviations are used in this manuscript:

CF	Cystic fibrosis
PwCF	People with cystic fibrosis
CFTR	Cystic Fibrosis Transmembrane Conductance Regulator
DNA	Deoxyribonucleic acid
MALDI-TOF-MS	Matrix-Assisted Laser Desorption/Ionization Time-of-Flight Mass Spectrometry
LB	Lysogeny broth
OD	Optical Density
NCBI SRA	National Center for Biotechnology Information Sequence Read Archive
ANI	Average nucleotide identity
MLST	Multilocus Sequence Typing
ICE	Integrative and conjugative element
IME	Integrative and mobilizable element
IS	Insertion sequence
VRNG1	Verona non-affiliated genogroup 1
SNP	Single-Nucleotide Polymorphism
Ts/Tv	Transition–Transvertion Ratio
AK	Aspartate Kinase
ATP	Adenosine Triphosphate
